# Metal Jewelry Craft Design Based on Computer Vision

**DOI:** 10.1155/2022/3843421

**Published:** 2022-06-15

**Authors:** Nan Li

**Affiliations:** Design College, Shandong University of Arts, Jinan 250300, Shandong, China

## Abstract

Combining computer vision technology with process design, a new design and production method is obtained, which breaks through the limitations of traditional jewelry creation and provides new possibilities for the realization of complex jewelry structures. When technology no longer becomes the bottleneck of artistic expression, the space of art will be greatly expanded. Science and technology leading design method has become a new way to assist jewelry artists in subjective creation. According to various thoughts and ideas in design, establishing the corresponding algorithm rules and parameters can generate the scheme through calculation. The design result obtained in this way not only has a scientifically logical basis but also obtains the result beyond the normal imagination space due to the intelligent design process. This paper tries to apply computer vision technology to modern jewelry design, analyzes several aspects of computer vision application in process design, and combines the latest technical means to put forward algorithms for verification. The results prove that computer vision can improve the efficiency of crafts design significantly.

## 1. Introduction

With the rapid development of the global economy and culture, mankind has entered an era of information explosion and rapid development of science and technology. A variety of information sources can impact people's senses through various media such as graphics and sound. In the modern society where productivity has been greatly improved, art has gradually been integrated into people's lives. Modern people are no longer only satisfied with the practicality of products but also have new artistic and ideological requirements [1]. It can be seen from the gold and silver jewelry brands and stores everywhere that the metal art embodied in metal jewelry has catered to the emotional and spiritual needs of most people [2].

In the 21st century, human beings are in a pluralistic information society. Culture and art have been different from the single form of the previous society. After the development of religious function and social functions in ancient times, the decorative function of jewelry has been gradually strengthened. Jewelry design and research not only cater to religious etiquette and reflect class wealth but also have become a conscious and controllable behavior gradually. Modern jewelry design and research pay more attention to the pursuit of decorative meaning, and this pure pursuit of decoration is also the development of humanities in different periods [3]. With a specific historical background, there are often costume cultural characteristics corresponding to it. After human society has developed to a certain extent, jewelry design reflects the most direct and pure decorative needs through certain research means. Modern jewelry design also needs effective research and development ways to excavate people's most urgent desire for jewelry decoration [4, 5].

Nowadays, the pace of life is getting faster and faster, and the rapid spread of Internet information makes the cultural industry face great challenges. Culture is also facing a crisis of convergence. In the field of jewelry design, jewelry products also lack cultural connotation and cannot realize cultural inheritance [6]. Many consumers with high quality and high artistic aesthetics are also eager to have jewelry works with far-reaching cultural connotations. Therefore, modern jewelry design and research need to pay attention to humanistic needs, use an effective research and development method to care about modern people's nostalgic mood, and explore people's inner intuition and desire for jewelry [7]. The most natural and pure decoration consciousness in people's hearts is the most direct display.

The rapid development of technology does not kill the art of metalworking but has encouraged the emergence of new industries. At present, the development of metal surface technology is led by the progress of science and technology [8], which has become the “processing type” metal surface technology. In the future, it is necessary to combine the “creative type” metal surface technology with brain and processing practice. Their mutual traction and influence can create works with more manual processing technology and modern machine production level. On the basis of studying the existing technologies, the metal surface technology will be carried forward and recreated, so that the metal surface technology can serve today's jewelry industry, humanities, and public aesthetics or provide materials and technology for the creation of artists [9].

Algorithm technology can tap the creative potential of designers and give designers more abundant imagination space. Computer technology plays an important role in the three-dimensional structure of the process and innovation, so this paper studies the application of jewelry design based on modern metal technology. We use a computer vision algorithm to analyze the different features of metal jewelry and further use network combination features to classify. After the primary classification of intelligent algorithms, new features are created in reverse through network algorithms, and these new features are applied to industrial models, so as to obtain the artistic products jointly created by machines and humans.

## 2. Related Work

Since humans started to smelt metal, they have begun to use metal to make all kinds of jewelry to decorate themselves. From the Stone Age to the use of simple and grinding tools to master the technology of smelting bronze and smelting silver to the blowing method [10] and then to the emergence of the smelting process, jewelry culture has experienced from grinding carved jade culture to bronze culture and the transition of precious metal gold and silver jewelry through attributes to various kinds of jewelry materials, design, and production. Historically, jewelry has an important status in clothing and ornaments. Ancient woman's crowns and jewelry are famous for their exquisite design at home and abroad. Men also pay attention to the role of jewelry seriously [11]. His jade pendants and hand-wearing-BanZhi are symbols of identity and grade. The delicacy of accessories not only reflects the superb craft level and technology but also represents the status of each class, so the ancient people attached great importance to the status of jewelry accessories.

The industrial revolution in Britain at the end of the 18th century brought about great social changes. Productivity rose dramatically, and handicraft workshops were replaced by large-scale mechanized production [12]. There was no doubt that the development of machinery aimed to serve human life. Labor force and time had been unusually saved and efficiency had also been improved. Cheap products were shipped all over the world. At the same time, the destruction of handicrafts brought by mechanized large-scale production was immeasurable. In the field of jewelry design, mechanized mass production was still the main production and development mode of jewelry products [13]. However, with the development of society and the improvement of people's aesthetic awareness, more and more consumers pursue the uniqueness of design, so limited sales and customization models came into being. Limiting the number of products and advocating unique design and research mode solved this problem.

At present, the demand for ornaments is growing, and the types are becoming richer and richer. People's pursuit of jewelry has not only stayed at the beautiful level but also seeks a sense of science and technology. The development of new jewelry and sensor is pluralistic and refined. The demand for creativity makes everyone more practical, so the development of science and technology application jewelry has certain progress and innovation at home and abroad. With the rise of the “Art Nouveau” movement, the choice of jewelry materials has also undergone great changes. Cheap and renewable materials have been widely used in jewelry production. These abundant simple materials with decorative functions have gradually replaced precious gold, silver, and gem materials that occupy an important position in the history of jewelry culture. Jewelry designers have also become clearheaded and slowly put their eyes on the beauty and touch of the material itself [14]. For jewelry design, computer-aided technology has come into people's sight. But in the early design, as the computer is expensive and complicated, much design work cannot be completed on the computer. Computer-aided technology only represents a new possibility and a new direction. With the rapid development and popularity of computer technology, more and more jewelry design software came into being to let computers become a common tool for jewelry designers [15].

At the present stage, one of the most widely used technologies is MBD (Model-Based Definition). Before this, technicians used two-dimension drawings to describe products and transfer information, which had great shortcomings [16]. At present, digital design and manufacturing technology is adopted to directly utilize the digital information data of information transmission, process design, and manufacturing mode based on a 3D digital model in product manufacturing [17]. The process information, tooling design, product quality inspection, and other information in the product can be obtained by the MBD model, which can shorten the research and development design cycle and improve the design quality of the products. However, for the model itself, due to its complicated three-dimensional model, the file occupies a large memory space, usually more than 100 MB or even several GB. MBD model has a large amount of information, which is not conducive to the acquisition of data information and the disadvantages of model anytime viewing due to a large amount of data when it is transmitted between different departments, such as slow viewing or slow information acquisition due to a large amount of data, or the failure of computer hardware to meet requirements [18]. In order to meet the demand for manufacturing, many scholars at home and abroad have done a lot of research studies on the lightweight of 3D digital MBD model data processing.

In the application of digital metal design and manufacturing, not only are 3D models used to replace the current 2D and 3D drawing conversion problems, but also design, process modeling, and simulation technology are used to improve the efficiency of product design and process production. For example, Wu et al. specially studied the application of complex shell MBD digital model in digital engineering in literature [19], compared four lightweight file formats, analyzed the advantages and disadvantages of the four formats, and verified their application in different application scenarios. Huang et al. introduced a method of extracting subcomponents from models in literature [20]. During the manufacturing process, effective parts of CAD models are retrieved to complete the reuse of 3D models. BalicJ et al. introduced a method to extract information from the model in literature [21] and studied the required functions of feature extraction of their CAD model based on the method of milling strategy prediction. The method adopted by Zhu et al. in literature [22] is to simplify the analysis by adding 3D model data information on the basis of a 2D model. Wang et al. studied the basis of the MBD model design in the literature [23], together with the present situation of the production process of soft and hardware environment; in the process of mechanical product manufacturing process model lightweight and visualization, 3D key technologies were analyzed, and the state of the extracted information into the feature information layer, three layers, and process chain used a recursive depth-first walk. The process information of constructing the lightweight model was extracted from the source point, the information file of the lightweight model was compressed, then the model was reconstructed to simplify the 3D model, and the visualization technology was used to realize the visualization of the 3D model.

With the deepening of research, more and more science and technology are introduced into metal design and manufacturing. People not only need to improve the process but also need to compute more flexibly and adopt light processing. In literature [24], Huang et al., in order to highlight the effect of display and reduce the amount of design data, adopted the method of artistic rendering to achieve this goal without the requirement of computer equipment. Although the sense of unreality of the artistic method can speed up the modeling, this method is operated on the original model. Although it achieves the effect of lightweight model data, the complete data information of the model is broken or even changed, which is not suitable for those applications requiring high model accuracy. The earliest Taubin and Ouma proposed a geometric lossless compression coding algorithm [25] based on grid processing, which is an efficient coding algorithm form that decomposes complex grid models into simple topological structure information compression coding, vertex prediction estimation, and compression. The grid model simplification algorithm is the most commonly used method to deal with the browsing and display of 3D models. Under the requirement of model accuracy, the number of triangular meshes can be reduced to the maximum. The most commonly used algorithms include surface merging, vertex deletion, and other mesh simplification algorithms [26]. Most of the lightweight processing of 3D MBD model data is aimed at the lightweight processing of ontology solid model, for the purpose of fast 3D browsing and visualization. A small amount of research on the lightweight processing of 3D MBD model data is in transmission and storage. At present, in the era of big data, there is almost no research on the lightweight transfer of MBD model data to local terminals in the cloud. With the rapid development of intelligent devices and sensors, technology represented by computer vision will continue to replace traditional metal design.

## 3. Intelligent Metal Jewelry Design

**Figure 1:**
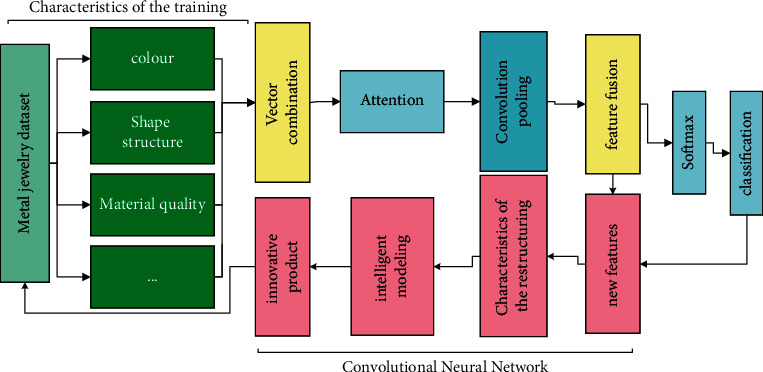
Network diagram of intelligent metal jewelry design.

With the change in people's ideology, the modern art design is also looking for various new forms of expression and development direction. With the advent of the information society, the design expression form accepted by people is no longer limited to a regional style. Considering the current cultural background and social background, on the one hand, the public's preference and aesthetic appreciation of modern design cannot be directly investigated by referring to the selection and evaluation criteria of traditional art. On the other hand, based on machine intelligence, people analyze metal jewelry data through computer vision, get a large number of metal jewelry features through training, and further classify them using the network. With the development of research, people reversely use the obtained features for recreation. Let network learning products and intelligent design meet human needs of modern products, and metal jewelry network flow based on computer vision is shown in Figure 1.

### 3.1. Traditional Metal Design

The traditional metal design method is the accumulation of human beings for thousands of years, which has an important position. The common forming process is mainly divided into forging, chiseling, welding, and casting [27].

1. Forging: the use of good ductility of metal, the metal plate after the high temperature is hammered plastic constantly, and finally made into relief, utensils, sculptures, etc.
2. Chisel: the use of the hammer, chisel, iron horse, and other tools on the metal surface carving pattern
3. Welding: it refers to the metal material pressure or heating, which can be used or not to fill the materials combined with the workpiece
4. Casting: the molten metal is injected into the mold and directly formed. Then, the surface process is treated

Traditional metal surface technology includes hammer forging, chisel, welding, gilding, silk inlay, hollowing, misplaced inlay, and gold and silver off. Ancient craftsmen expressed the texture of the metal surface in different ways through various metal surface processes. In an excellent process, designers can often dig out the biggest characteristics and natural beauty of the material itself through various processes. The following is a brief introduction to some common traditional crafts.

Welding process: the forming method of the metal bead is to cut the metal sheet into small sections through high-temperature heating to make it fuse into granules naturally. The welding process is to heat and melt the metal beads and fuses them onto the surface of the metal product directly, forming a pattern composed of gold beads. Compared with the welding method using solder, the advantage of molten metal particles is that the welding area is small and the performance effect is clean.

Gilding process: gilding is a mixture of pure gold and mercury plated on bronze ware, which is called “gold amalgam.” Since gold is dissolved in mercury and can flow freely, these two things boil at different points. Heating vaporizes the mercury, leaving the gold to adhere to the surface to form gold patterns.

Silk inlaying process: silk process is to process gold or pure silver into the silk of different thicknesses for bending, pinching, filling, stacking, and other operations and the production of gold and silver jewelry fine gold process. Different decorative parts can be made into different patterns of silk, such as arch silk, bamboo silk, and wheat silk, and other shoulders can be welded to the shoulder of gold and silver utensils, becoming independent decorations.

Gold foil process: because of the good ductility and plasticity of gold, it is hammered into thin slices for pasting Buddha statues, utensils, etc. This craft is the traditional craft of the Chinese nation, which has a history of nearly 1700 years. The unique gold foil forging technique born in Nanjing was listed as the first batch of national intangible cultural heritage by The State Council.

Gold sticking process: gold sticking process is a traditional process with the emergence of gold foil. It has the same meaning as modern gold coating technology. That is, the use of the adhesion of very thin gold foil, the mutual absorption of some smooth surface materials, and the gold foil package affixed to the surface of the utensils play protective and decorative roles.

Gold coating process: the gold foil is coated on the surface of copper, aluminum, zinc, iron, or other alloy materials and hammered solid so that the gold foil is attached to the surface of the body and the formation of concave and convex texture is consistent with the surface of the body to play the role of decoration and protection. The gold foil used for coating gold is usually gold with higher purity.

Gold and silver off process: it is developed by the Shang dynasty gold foil decal process, which is a famous ancient Chinese decoration process. Usually, the gold foil and silver foil are processed to the desired pattern and then coated in layers of paint and ground until the gold and silver reveal the corresponding pattern.

Misplaced gold and silver: chiseling and carving shallow grooves of patterns in the surface of the metals. Then, gold and silver wires, pieces, or beads are wedged into the groove, finally, beating and using grindstones to make them flat. This is a new development of the traditional bronze Mosaic technique. The gold and silver leveling and stripping technology used for lacquer surface decoration belong to a special form of gold and silver splicing technology.

Dotted jade craft: dotted jade craft is a traditional Chinese jewelry-making craft. It is one of China's national treasures along with gilding and enamel. First, gold or gold-plating metal is used to make different patterns of the base. Then, the bright blue kingfisher back feathers are carefully inlaid on the table to make a variety of jewelry. After this, the color of the jewelry is bright and will never fade [28].

### 3.2. Modern Metal Working Based on Computer Vision

#### 3.2.1. Metal Forming Technology

With the continuous improvement of the modern industrial level, the requirements of material production scale, production reliability, and production quality are also improved. In this context, it is imperative to achieve the automatic production of materials. At the same time, the automatic production of materials can also effectively solve the problems of manual operation errors, low production efficiency, and low prominence and ultimately achieve the purpose of improving production efficiency and ensuring product quality.

Machine tool intelligent investment casting: it is the use of CNC machine tools according to computer vision 3D modeling or existing products directly to the metal cutting, grinding, and other processes. Due to the technical limitations of machine tools themselves, such as less axial direction, tool selection, and other reasons, machine tools are generally used to produce products whose shapes are combined on the basis of basic geometry.

3D printing technology: 3D printing is the crystallization of computer vision highly applied technology, that is, a kind of rapid prototyping technology by using powdery metal or plastic and other adhesive materials with layer printing and stacking model structure. Later, 3D printing technology has gradually matured and finished products can be directly printed by 3D printers.

Science and technology and traditional craftwork must be combined to achieve better development. With the progress of technology, many excellent and exquisite metalworking techniques have been lost due to various reasons, but the retained techniques are still worth learning and studying by modern metal artists. For example, forging, chisel, welding, gilding, filaments inlaid, hollowing, inlaid wrong, and gold and silver off in modern metal art design can still be referred to for reference and application. Its practical application is also in metalworking teaching practice in colleges and some personal metal studios.

#### 3.2.2. Metal Surface Technology

Modern intelligent metal surface treatment technology is of great significance. The intelligent surface treatment technology greatly improves product quality and saves valuable materials, realizing material surface compounds. Solving problems cannot be solved by a single material to repair the overall advantage, saving energy and materials significantly.

Polishing process: it is the use of a polishing machine to complete the polishing process. When the polishing wheel rotates at high speed, high temperature is generated between the jewelry and the polishing wheel as well as the molten polishing wax, which improves the plasticity of the metal, the surface fine unbalances, and the brightness of the jewelry. If the surface of the material is rough, it is difficult to plate a solid and corrosion-resistant coating. Even if the coating is barely on, the jewelry coating will exuviate, bubble, pit, speckle, and has other undesirable phenomena in a short time. Therefore, the workpiece must be polished before electroplating. Metal polishing based on computer vision, using the algorithm to calculate the time and strength in advance, reduces the loss of materials. And it can accurately control the completion of the process.

Electroplating process: after the precious metal jewelry workpiece is processed and formed, the surface color is its inherent color. However, sometimes, it is necessary to change the surface color of the metal jewelry to achieve a special effect. After the object is polished, processed, and electroplated before plating, the electroplating coating is formed, which makes the surface luster of the object strong. The main purpose of electroplating is to enhance the corrosion resistance of the metal, increasing the appearance and surface hardness. Electroplating is a chemical surface treatment process. It is prepared according to the requirements of jewelry electroplating special gold-plating solution in a certain PH value and temperature conditions through the electrochemical reaction between positive-negative electrode current and electroplating solution so that the gold ions of the gold-plating solution are gradually transferred to the metal surface of the jewelry.

Electrocasting is a new jewelry production process. Its principle is similar to electroplating, and casting in the solution is negative mold whose surface activation is treated after the generation of the conductive layer. Through the action of electrophoresis, metal will be gradually deposited on the negative mold surface, and the thickness can be removed after grinding and welding. This surface treatment is electrocasting the jewelry. This kind of jewelry has a beautiful appearance, large size, lightweight, fast electroforming speed, and high or low output, which is easy to master flexibly. At present, gold ornaments on the market such as mascots, Buddha, and zodiac are mostly hollowed into gold ornaments by electrocasting.

Sandblasting process: the metal surface is sandblasted according to the design requirements so that the metal surface shows the frosted texture. Compared with other surface textures, it enhances the artistic performance of the product and has increased its aesthetics.

Imprint process: it is also known as indentation, which is a decorative technique to produce texture properties on the metal surface. It is a process that makes the pattern needed to be designed into a mold and presses out the texture on the surface of the metal through stamping by using the characteristics of metal molding and ductility. Impression can only be made on one face of the metal plate. The texture of each imprint is mainly determined by the chisel.

Wood grain metal surface process: wood grain gold process is derived from the traditional Japanese sword-making technology, which is a metal-making process studied by sword casting division when forging swords. Swordsmith uses layers of metal in an attempt to mimic the ancient Chinese lacquer technique of stacking vermillion. Lacquers overlay layers of paint on vessels and, then, carve curving grooves on the surface of tang grass or swirls to reveal a continuous pattern of stripes of various colors. Grain gold is called “grain metal” because the visual effect of the metal layer is similar to the grain of wood.

Enamel process: enamel process is a unique combination of porcelain and metal processes. In the polished metal substrate surface, it is coated with a layer of glass gloss enamel. After drying and burning, it becomes a magnificent and colorful craft. It not only has the characteristics of precious and solid metal but also has the characteristics of crystal clear enamel glaze, which is smooth and suitable for decoration. Enamel works mainly include painting enamel, wire drawing enamel, hammer forging enamel, and tire enamel.

### 3.3. Generative Adversarial Network

#### 3.3.1. Metal Jewelry Image Processing

*(1) Image Denoising*. The purpose of image denoising is to remove the noise while preserving the important signal features as much as possible. In the process of image acquisition, there are many noises and interferences. Noise can be understood as the interference signal that hinders the process of image observation and information extraction. Noise may be introduced in every link of image generation and transmission, among which additive White Gaussian noise and salt and pepper noise are more common. The interference of the image noise irreversibly destroys the information of the image. The image noise model can be roughly approximated as

(1) $$\begin{array}{c} f\left(x\right)=u\left(x\right)+n\left(x\right), \end{array}$$

where *u*(*x*) represents the real signal value, and *n*(*x*) represents the noise at position *x*.

The mean filter replaces the value of the window center with the average value of the pixel values covered by the window. The existence of noise causes a sharp gray difference in the image at the noise point, and it is this gray jump that leads to the observation obstacle. Eliminating the noise through the neighborhood method will also lead to the blurring of the edge part with the same sharp gray difference. Suppose the image is *f*(*x*, *y*), and the image after mean filtering is defined as

(2) $$\begin{array}{c} \hat{f}\left(x,y\right)=\frac{1}{mn}\sum_{\left(s,t\in S_{xy}\right)}f\left(s,t\right), \end{array}$$

where *m* and *n* are, respectively, the height and width of the filter window, *s*_*xy*_ represents the location set of all pixels in the window, and *s* and *t* are the locations of pixels in the window.

A median filter provides excellent noise reduction capability for some types of random noise, which can effectively smooth sharp noise and better retain an edge, and it is often used to filter salt and pepper noise. To a certain extent, isolated noise can be distinguished from image features that need to be preserved, such as image edges and lines.

*(2) Image Enhancement and Morphological Processing*. Image enhancement enhances the discrimination ability of some information according to specific needs while weakening or removing some unwanted information interference so that the processed image is more convenient for the subsequent processing links and more suitable for the current task needs.

Histogram equalization is a common technique to enhance image appearance. For an overall dark image, its histogram will be tilted toward the lower end of the grayscale. If we can “extend” the gray level of the dark end so that the gray value is evenly distributed in [0, 255], the gray-level difference between the background and the object in the image will be enlarged. Thus, it will increase the contrast of the image and make the image clearer. It can be found that there are scratches and macula defects on the surface of metal products. If the original image is directly used for defect detection, macula defects are not obvious and it is not easy to detect defects. Through image enhancement, the defect areas are highlighted. Morphology is mainly used in image preprocessing, enhancing object structure, and adjusting shape features, and it is widely used in image analysis. Corrosion and swelling are basic morphological changes based on which more morphological operations can be defined.

*(3) Image Segmentation*. Image segmentation is a prerequisite for image processing. Through image segmentation, the image is divided into regions with different features, and the region of interest is extracted. Usually, it can be segmented by grayscale, texture, color, and other features. There are many methods and types of image segmentation, among which the threshold-based image segmentation method is suitable for grayscale images with a large grayscale difference between target and background, which is simple and quick, so this paper mainly adopts threshold-based segmentation. The key to threshold segmentation lies in the determination of the threshold and a good segmentation method is conducive to the identification and analysis of subsequent targets.

The least mean square error method is one of the commonly used threshold segmentation methods, where *z* represents the gray value and *P*(*z*) is the probability density estimation of the gray value. Two probability density functions *P*_1_ and *P*_2_ are defined, corresponding to the gray values of background and foreground, respectively. Then, the mixing density function of the whole image is

(3) $$\begin{array}{c} p\left(z\right)=p_{1}p_{1}\left(z\right)+p_{2}p_{2}\left(z\right),\\ p_{1}+p_{2}=1. \end{array}$$

So, the pixels in the image are divided into foreground and background. The aim of the least mean square error method is to minimize the probability of prediction error when selecting the threshold *T*. Based on computer vision, metal products are generally divided into three regions, namely, texture region, smooth region, and outer circle region, so it is defined as

(4) $$\begin{array}{c} F\left(x,y\right)=T\left(x,y\right)+S\left(x,y\right)+C\left(x,y\right), \end{array}$$

where *F*(*x*, *y*) is the original image, *T*(*x*, *y*) represented the texture region image, *S*(*x*, *y*) is the smooth region image, and *C*(*x*, *y*) is the outer circle region image. The texture area is special. The surface is not smooth but has certain texture characteristics, which needs to be extracted separately for special treatment. The smooth area of normal metal products is relatively flat. For the smooth area of different products, the gray value trend of normal products is flat while defective products fluctuate. This area can be extracted separately to make better use of this advantage. In this paper, two ring masks are used to separate the texture region, smooth region, and outer circle region.

Setting different threshold values for different regions can reduce the adverse effects of uneven illumination, which is easier to feature extraction of the metal products. For instance, if metal product surface polishing on the right side is bright, and the mean gray level is higher than the left, then setting the threshold can appropriately increase (such as using the grayscale average as discriminant conditions, A compared to B in the region, and gray-level threshold can be appropriately increased). Similarly, on the left side of the metal product, the gray mean value is low, so the set threshold can be appropriately reduced.

#### 3.3.2. Computer Vision Metal Surface Classification

Traditional image classification is often aimed at a specific recognition task, with small data scale and poor generalization ability. It is difficult to achieve an accurate recognition effect in practical applications for huge image data and serious image interference. In addition, traditional image processing methods often require complex threshold settings for defect recognition, which are sensitive to environmental factors such as illumination conditions and background. If environmental factors change, the settings of these thresholds need to be carefully adjusted; otherwise, the algorithm cannot adapt to the new environment and will lack adaptability and robustness. In feature extraction, it is easy to ignore or fail to understand some complex, hidden, or nonintuitive phenomena and ignore some feature variables depending on researchers' prior knowledge and cognition of classification tasks, while the extracted features directly affect the performance of the system.

In feature extraction, deep learning can automatically extract higher-dimensional and more abstract features from raw data instead of complicated manual feature description and extraction. In contrast, in the field of computer vision, a network model based on deep learning has a stronger ability for feature learning and feature expression. Neural networks can reveal more features that affect the quality of recognition in a positive way and build more general and accurate recognition methods. CNN performs convolution operation on the input 2D image through a series of convolutions with adjustable parameters and further convolves the obtained results to form a cascade, so as to realize the localization of network connection and reduce the number of parameters through weight sharing. A cascaded neural network can extract pattern features of different levels in the input image by gradually expanding the perception domain, which can be used to process different types of tasks.

*(1) Convolution Layer*. The convolutional layer extracts different hierarchical features of the input image. Each element of the convolution kernel is constantly adjusted by the error backpropagation method during training. The convolution operation realizes local connection and weight sharing through sliding Windows, which increases the translation invariance of images and is conducive to better generalization performance, and can greatly reduce the number of parameters compared with multilayer perceptron.

Assuming that the input size is (*H*, *W*), the filter size is (*FH*, *FW*), the output size is (*OH*, *OW*), the filling is *P*, and the step is *S*, we calculate the values of *OH* and *OW* as follows:

(5) $$\begin{array}{c} OH=\frac{H+2P-FH}{S}+1,\\ OW=\frac{W+2P-FW}{S}+1. \end{array}$$

The pooling layer can achieve three functions: feature invariance, feature dimension reduction, and overfitting prevention. Concerning the average (maximum) pooling during each operation, the average (maximum) of the area covered by the pooling core is used as the pooling result.

The function of activation is to introduce nonlinearity into the network and determine how to activate the sum of input signals. The sigmoid function used to play an important role in the development of neural networks, but it will produce a gradient “saturation effect.” Relu is one of the most commonly used activation functions in deep convolutional neural networks. The Sigmoid function is defined as

(6) $$\begin{array}{c} \sigma \left(x\right)=\frac{1}{1+\exp\left(-x\right)}. \end{array}$$

Relus is considered to have more biological characterization, which can yield more favorable results for image recognition tasks because the function is less susceptible to the vanishing gradient problem and can produce a sparser and efficient representation. The Relu function is defined as

(7) $$\begin{array}{c} \max\left\{0,x\right\}=\left\{{}_{0,x<0.}^{x,x\geq 0,}\right.. \end{array}$$

*(2) Full Connection*. For image classification problems, the full connection layer is connected at the end of the network, used to vectorize the feature map, and used this vector to calculate the classification results. In the last few layers of convolution and pooling of the network, high-level semantic information of the image has been extracted, which exists in the feature map. Vectorization of the feature map can be considered to obtain the feature vector of the source image. The fully connected layer has a large number of parameters, and a small number of computing layers involved in the final decision adopt the fully connected structure.

## 4. Application Simulation Experiment

### 4.1. Data Preparation and Evaluation Indicators

Since computer vision requires a large amount of data support, and metal jewelry design covers trade secrets and copyright, we choose Kolektor Surface Defect Dataset. Traditional machine learning can usually learn a model based on a given sufficient training data and then use that model to make predictions. In order to reduce the interference of invalid regions on feature extraction, improve the training speed, and reduce memory consumption, the input object is processed to some extent, and only the measured object is retained in the final effect. The data set was randomly divided into training data set, test data set, and verification data set in the ratio of 8 : 1 : 1. Experimental data preparation is shown in Table 1.

The training set is used to fit the classifier parameters, and the verification set is used to adjust the classifier parameters. In the training stage, the test set is encapsulated, and in the final stage, the generalization ability of the trained model in practice is estimated by the test set. At the same time, the sample size is expanded by means of data augmentation. The system mainly adopts random horizontal flip, random vertical flip, and random rotation of 180 degrees.

In order to evaluate the performance of the metal surface classification system based on neural network architecture in practical application, the commonly used evaluation indexes for multiclassification tasks mainly include accuracy (Acc), precision (*P*), recall (*R*), *F*1-score, and confusion matrix. In classification tasks, accuracy, also called precision, is the most commonly used index. Accuracy refers to the ratio of the number of samples correctly predicted to the total number of samples participating in the prediction. Corresponding to accuracy, the evaluation index is the error rate, which refers to the ratio of the number of samples that are wrong in prediction to the total number of samples that participate in prediction. Both measure the prediction of the global sample.

(8) $$\begin{array}{c} \text{Accuracy}=\frac{TP+TN}{TP+TN+FP+FN},\\ \text{Precision}=\frac{TP}{TP+FP},\\ \text{Recall}=\frac{TP}{TP+FN},\\ F1=\frac{2\times \text{Precision}\times \text{Recall}}{\text{Precision}+\text{Recall}}. \end{array}$$

### 4.2. Experimental Verification

Metal jewelry data is different from the conventional computer vision data set. In order to make the verification results more authentic, we choose transfer learning. It can migrate a large network trained on a large data set to a small data set so that good results can be achieved only with a little training. To some extent, transfer learning increases the utilization rate of the model and solves the problem of missing data, which is exactly what metal jewelry needs. In order to verify the network model based on CNN improved pretraining proposed in this paper, we simultaneously analyzed several mature models, VGG19, ResNet, and DenseNet, fine-tuned their structures, migrated to the local metal surface in the way of pretraining, and made the score as close as possible to the real metal jewelry.

#### 4.2.1. Basic Experiment Preparation

Before the loss change experiment, the basic parameters of training need to be set. For the SGD optimizer, good performance can be achieved when the initial learning rate is 0.001. The best performance is achieved when the momentum decay factor is 0.9. For Adam optimizer, better performance can be achieved when the initial learning rate is 0.0001. When the momentum attenuation factor is 0.8, the performance can be significantly optimized. For the RMSProp optimizer, better performance can be achieved when the initial learning rate is 0.0001. However, the training process and verification process corresponding to the optimizer fluctuate greatly, so it is not recommended to be used in the current metal surface defect classification system. After several tests, the SGD optimizer was finally selected, with a learning rate of 0.001 and a momentum decay factor of 0.9.

The learning rate also directly affects the efficiency of the network model, and the performance of the system in the practical application can be measured by its performance in the validation set. In terms of accuracy and loss function, the system performs worst when the learning rate is 0.05. When the learning rate is 0.001, the system has the best performance, which is close to the performance when the learning rate is 0.0005 and can achieve the best accuracy and minimum loss function value on the verification set. Therefore, the final learning rate is determined to be 0.001.

In the training stage, the topological structure and hyperparameters of the transfer module can remain unchanged. According to whether the weight parameters are retrained according to the new data set, the transfer learning mode can be divided into pretraining mode and fixed-value mode. In the pretraining mode, the migrated weight is regarded as the initial weight of the new network, and the value will be changed by the gradient descent method during the training process. In this way, not only can the migrated knowledge be retained but also flexible adaptability can be ensured so that the migrated knowledge can be flexibly adjusted through the training of the new network on the new data. In contrast, in the fixed-value mode, the structure and weight of some migrated networks remain fixed, and the training process is only targeted at the fully connected network behind the migrated modules. Therefore, there are fewer parameters to be adjusted, and the learning convergence speed is faster. The performance of the two training methods using transfer learning is better than that of the training method without transferring in terms of accuracy and loss function value, and the epoch value is 50.

#### 4.2.2. Loss Function

**Figure 2:**
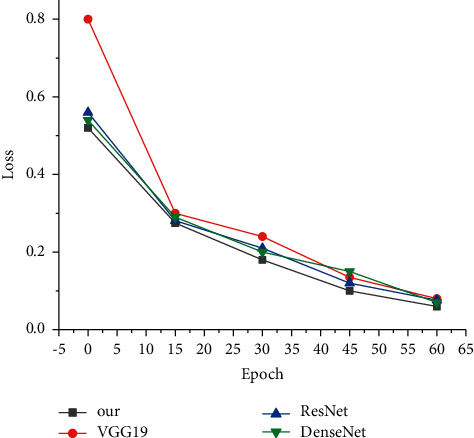
Comparative experiment of loss function change.

The improved CNN architecture in this paper is due to its lower computation and parameter quantity than other network models while ensuring accuracy. For different network architectures, we compared VGG19, ResNet, and DenseNet. In the comparative experiment, the pretraining mode of transfer learning was uniformly adopted to fine-tune the network architectures. As can be seen from Figure 2, the improved model based on the pretrained CNN network has the best performance. On the one hand, the loss starting point is low and the overall curve is flat; on the other hand, the model in this paper is stable when the epoch is 50. Among them, the VGG model changes greatly and may have the problem of a huge model. The loss changes of ResNet and Dense Net models are very similar, but the overall results are still not as stable as the model in this paper.

#### 4.2.3. Model Computation

In order to further verify the quality of the model, we verified the efficiency of the model mainly through model parameters and model computation, as shown in Table 2. By comparison, it is found that it is difficult for these models to achieve a high recognition rate with less model parameters and less model computation, and the recognition rate is always proportional to the complexity and computation.

From the perspective of the number of model parameters and the amount of model calculation, the number of model parameters and the amount of model calculation of the ResNet network model are 22M and 7000M, respectively, which are lower than the number of parameters and the amount of model calculation of the model in this paper, which are more conducive to the application of embedded terminals and other platforms. However, the traditional model has the worst performance in the accuracy of the verification and training phase, and it is difficult to meet the actual needs of the industry in the accuracy of basic metal surface recognition. VGG network architecture in the verification stage, the number of parameters, and the amount of computation corresponding to the network architecture are 140M and 35000M, respectively, which is obviously not suitable for the deployment of embedded terminals.

#### 4.2.4. Accuracy

**Figure 3:**
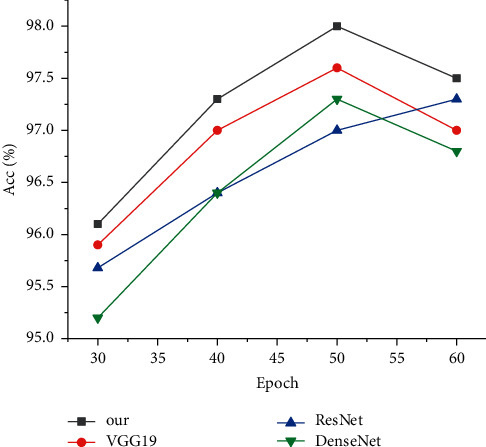
Accuracy comparison experiment.

The accuracy rate directly reflects the quality of the model and determines whether the model can adapt to practical application, as shown in Figure 3.

As can be seen from the experimental results, the model in this paper has been ahead of other methods at the beginning of training, but unfortunately DenseNet model is significantly lower than other models. With the progress of training, ResNet and DenseNet were getting closer and closer and basically reached a flat state in 40epoch after the DenseNet surpassed them. For the variation curves of the model in this paper, VGG19 and DenseNet were very similar, reaching the peak around 50epoch and beginning to decline afterward. ResNet requires a longer training time and slower model response.

#### 4.2.5. Other Indicators

**Figure 4:**
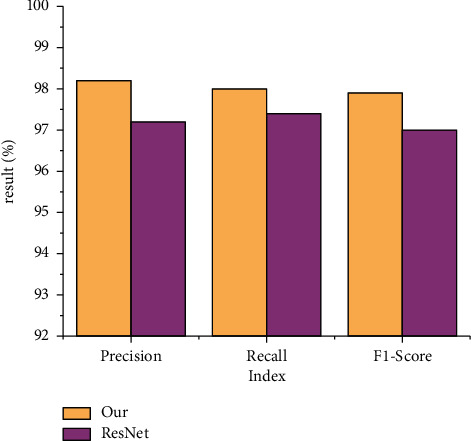
Comprehensive experimental index comparison.

Whether the computer algorithm can be applied to metal design needs to be considered in many ways, not only testing accuracy but also other indicators. The metal surface recognition and classification system can correctly identify test data, or the phenomenon of the wrong classification can occur. Even if the metal surface is close to each other, its performance is still better than the metal surface detection system based on traditional methods. Due to various factors such as oxidation, scratches, and impurities in the actual production, distinguishing is also difficult to quantify, because with the passage of time, the scratches are easily transformed into impurities, black points, or line even areas, leading to two defective characteristics of overlap, as shown in Figure 4 in this paper, the model of various other indexes.

## 5. Conclusion

Using computer vision to process design is a new means and thinking mode of computer-aided design, which provides a new possibility for modern jewelry design. Artificial intelligence technology provides a richer source of inspiration for jewelry, and jewelry also speaks for science and technology as a new medium. Jewelry designers only need to formulate and select algorithms according to the leading factors and then carry out the calculation through the computer; the results will be far beyond the designer's imagination. For modern jewelry, innovation based on traditional design methods is very important. As an auxiliary design tool, the participation of algorithm technology is positive for the design and production of modern jewelry. In this paper, it is proposed that computer vision technology can be applied to modern jewelry design, and a detailed theoretical analysis has been made to prove the necessity and feasibility and to provide new ideas and new methods for jewelry design. It extends the original thinking limitations by relying on the computer ability for data analysis and graphics generation and enriches the possibility of jewelry.

## Figures and Tables

**Table 1:** Preparation of experimental data.

	Scratch	Normal	Crack
Train	400	200	240
Verify	50	25	30
Test	50	25	30
Total	500	250	300

**Table 2:** Model parameters and calculation.

Model	Parameters	MAdd
Our	2M	600M
VGG19	140M	35000M
ResNet	22M	7000M
DenseNet	7M	5500M

## Data Availability

The experimental data used to support the findings of this study are available from the corresponding author upon request.
